# Grapheme-Phoneme Learning in an Unknown Orthography: A Study in Typical Reading and Dyslexic Children

**DOI:** 10.3389/fpsyg.2018.01393

**Published:** 2018-08-15

**Authors:** Jeremy M. Law, Astrid De Vos, Jolijn Vanderauwera, Jan Wouters, Pol Ghesquière, Maaike Vandermosten

**Affiliations:** ^1^School of Interdisciplinary Studies, University of Glasgow, Glasgow, United Kingdom; ^2^Parenting and Special Education Research Unit, KU Leuven, Leuven, Belgium; ^3^Laboratory for Experimental ORL, KU Leuven, Leuven, Belgium

**Keywords:** dyslexia, literacy, phonological awareness, orthographic knowledge, letter-speech sound learning, grapheme-phoneme-correspondences, artificial script, children

## Abstract

In this study, we examined the learning of new grapheme-phoneme correspondences in individuals with and without dyslexia. Additionally, we investigated the relation between grapheme-phoneme learning and measures of phonological awareness, orthographic knowledge and rapid automatized naming, with a focus on the unique joint variance of grapheme-phoneme learning to word and non-word reading achievement. Training of grapheme-phoneme associations consisted of a 20-min training program in which eight novel letters (Hebrew) needed to be paired with speech sounds taken from the participant's native language (Dutch). Eighty-four third grade students, of whom 20 were diagnosed with dyslexia, participated in the training and testing. Our results indicate a reduced ability of dyslexic readers in applying newly learned grapheme-phoneme correspondences while reading words which consist of these novel letters. However, we did not observe a significant independent contribution of grapheme-phoneme learning to reading outcomes. Alternatively, results from the regression analysis indicate that failure to read may be due to differences in phonological and/or orthographic knowledge but not to differences in the grapheme-phoneme-conversion process itself.

## Introduction

The ability to encode and decode meaning by using a collection of distinct markings, or simply put, the ability to read and write, is an impressive accomplishment. Unlike spoken language, which in the general population can be mastered without direct instruction, explicit instruction over a period of several years is required to master the ability to read and write.

Most children are capable of acquiring adequate grapheme-phoneme knowledge within the first year of reading instruction. However, the achievement of full automatization and integration of separate phonemes and graphemes into a single audiovisual unit (also known as grapheme-phoneme binding), requires several years of literacy experience and requires the formation of a functional neuro-circuitry converging print and spoken language processing networks, permitting a child to emerge as a skilled reader (Blomert and Vaessen, 2009; Blomert, 2011; Holloway et al., 2013; Clayton and Hulme, 2018). Since it has been theorized that a disruption in the development of integrated grapheme-phoneme representations interferes with the acquisition of accurate and/or fluent word reading, our research will investigate how these grapheme-phoneme mappings are learned in typical and dyslexic reading children and what the unique contribution to reading is.

In developmental dyslexia, present in 5–7% of children, reading is nearly insurmountable, in the absence of any motivational, perceptual or environmental explanation (Snowling, 2000). It has been proposed that the poor decoding abilities observed in people with dyslexia stem from a cognitive deficit in the development of, and/or access to, phonological representations leading to a difficulty in the processing of sounds in oral language (Snowling, 2000; Boets et al., 2013). The ability to attend to and manipulate speech sounds is essential for the formation and automatization of grapheme-phoneme correspondences which in turn is the foundation of accurate and fluent decoding. Research across various age (Shaywitz et al., 2007) and language groups (Ziegler and Goswami, 2005) has provided support for the phonological deficit theory of dyslexia and transparency of the language. Furthermore, the observation of the existence of a phonological deficit prior to the onset of formal reading instruction, and its significant relation to later literacy achievement, has lent support for the phonological deficit's potential causal role in dyslexia (Wagner and Torgesen, 1987; Pennington and Lefly, 2001; Boets et al., 2011; Snowling and Melby-Lervåg, 2016; Law et al., 2017b; but see Castles and Coltheart, 2004) for a critical review on causal evidence based on longitudinal studies).

Although phonological deficits have been demonstrated to account for a significant portion of the variance in reading by dyslexics, a significant portion of the variance remains unexplained. To account for this remaining variance recent studies have begun to explore alternative cognitive variables which may contribute for the literacy difficulties of individuals with dyslexia; such as orthographic processing (Ziegler et al., 2010; Boros et al., 2016) morphological awareness (Law et al., 2015, 2018; Cavalli et al., 2017) or statistical learning (Krishnan et al., 2016; Schmalz et al., 2017, Vandermosten et al., 2018). An additional factor that has been proposed to act as a critical bridge between the previously identified cognitive and behavioral deficits of individuals with dyslexia lays within the formation and integration of grapheme-phoneme mappings. Past research has theorized that deviant grapheme-phoneme integration of individuals with dyslexia is a result of phonological problems (Snowling, 2000). However, Blomert and Willems (2010) observed difficulties in grapheme-phoneme integration that were independent of phonological difficulties. Furthermore, brain potential and neuroimaging research comparing neural activation of dyslexic and typical reading participants also support the hypothesis of less integrated grapheme-phoneme representations in poor readers. Such studies have demonstrated that individuals with dyslexia exhibit reduced activity in response to grapheme-phoneme associations in the superior temporal sulcus region of the brain, a region highly associated with neural integration of grapheme-phoneme pairs. However, the same individuals with dyslexia were found to demonstrate adequate knowledge when graphemes and phonemes were separately presented. These results suggest a problem in the automatic integration (Froyen et al., 2009; Blau et al., 2010; Blomert, 2011; however, see Nash et al., 2017). Furthermore, in a study by Froyen et al. (2011), 11-year-old children with dyslexia exhibited brain responses which did not show any evidence of letter-speech sound integration and were noted to be comparable to the weakly associated effects found in typical first-grade readers.

Although these studies provide evidence supporting the presence of letter-speech sound learning deficits in dyslexia, the learning process of integrating graphemes and phonemes is never monitored as these studies use native graphemes and phonemes that are already trained for years. When using native phonemes and graphemes the differences in grapheme-phoneme coupling might be driven by prior letter knowledge and the degree of exercise and reading experience. In that perspective, the behavioral study by Aravena et al. (2013, 2017) and Karipidis et al. (2018) differed from past studies in its testing of newly learned association through the use of a novel script. The use of an artificial orthography, in contrast to the use of the subjects' native orthography, allows for a characterization of the initial learning process involved in the creation of grapheme-phoneme associations, in contrast to a mere assessment of the product of this learning. The use of such a novel script permits the control over conditions relating to how the associations were formed, such as length of exposure and instruction methodology.

In the studies by Aravena et al. (2013, 2017), children had to learn eight basic grapheme-phoneme correspondences using unfamiliar Hebrew letters and speech sounds derived from the participants' native language (Dutch). Results from Aravena et al. (2013) study indicated that the basic knowledge of the newly learned grapheme-phoneme associations was mastered equally well by both typical and dyslexic readers. However, dyslexic children performed worse on a word reading task within an artificial orthography. These results led Aravena et al. (2013) to conclude that the process of letter-speech sound binding is impaired in dyslexia. In a follow up study by Aravena et al. (2017) typical readers were eventually found to outperformed the dyslexic readers for accuracy and speed on a letter–speech sound matching task, thus supporting their initial claim. However, the inconstancy between the studies raises question concerning the nature of these observed differences in performance. It could be argued that if a phoneme-grapheme binding deficits are present within readers with dyslexia, it would be expected to be observed during the assessment of newly learned letter-speech sound association across both studies and not only be observed as a deficit in reading when using these newly learned associations which partly relies on phonological skills, as reported in Aravena et al. (2013).

Building on these concerns, Peterson and Pennington (2015) argued that the interpretation of these results is ambiguous, as results could be a function of the unimodal phonological deficit of individuals with dyslexia and not unique to a letter-speech sound binding deficit *per se*. The cross-modal integration, required to form accurate audiovisual representations of letter-speech sound correspondences, is complex and involves two very different representational systems, namely phonological and orthographic representations (Litt and Nation, 2014; Clayton and Hulme, 2018). Thus, the observation of dyslexic reader's failure to accurately integrate letter-speech sound correspondences could be attributable to a failure of phonological and orthographic processes in isolation or an issue directly concerning the association process itself. For instance, research has demonstrated the existence of early problems in phonological processing, before the introduction of print of children later found to have dyslexia. These early problems with phonological processing have been shown to be a strong predictor of later reading via measures of letter naming, rapid naming, morphological awareness, and phonological awareness (Boets et al., 2007; Torppa et al., 2010; Law et al., 2017a,b).

Therefore, any future work exploring letter-speech sound binding deficits would need to control for unimodal phonological processing and orthographic processing across groups. Such a design would aid in reducing any ambiguity and permit an assessment of letter-speech sound binding deficits and their independent contribution to reading (Peterson and Pennington, 2015).

Although the Aravena et al. (2017) included measures of phonological awareness as controls, orthographic processing controls were not included when exploring how letter-speech learning contributions to predicting individual differences in reading and spelling ability. An issue this study will set out to address.

### The present study

To address this gap in the literature, the current behavioral study is set out to examine letter-speech sound learning of individuals with dyslexia while controlling for unimodal phonological and orthographic processing across groups. The aims of this study were two-fold. First, we examined whether letter-speech sound binding deficits of individuals with dyslexia were behaviorally detectable within the initial stages of learning an unfamiliar script. Secondly, we examined the relationship of letter-speech sound learning in individuals with and without dyslexia with phonological and orthographic processing and its independent contribution to reading outcomes. Since the majority of the previous studies reviewed above found letter-sound binding deficits in dyslexics (i.e., Blomert, 2011; Aravena et al., 2013, 2017; with the noted exception of Nash et al., 2017), we hypothesized that such deficits would be observable within our dyslexic group. Additionally, we hypothesize that the ability to learn letter-sound connections would be a good predictor of reading, independent of phonological awareness (Blomert and Willems, 2010; Aravena et al., 2017), orthographic skills and naming speed.

To achieve these ends, this study used the same implicit learning task and assessment procedure as that used by Aravena et al. (2017). Children with dyslexia and typical readers were provided with a short computer game based training program, aimed at the learning of 8 basic letter-speech sound correspondences within an unfamiliar script (Hebrew), paired with familiar phonemes derived from their native language (Dutch), thus allowing for an assessment and comparison between the groups of the initial phase of learning a novel script. Additionally, an assessment using an artificial script paradigm allows for the control of differences in previous exposure to experimental stimuli, thus more closely mimicking the early phase of learning to read in children irrespective of prior linguistic knowledge.

## Methods

### Participants

All children were recruited from the longitudinal study reported by Vanvooren et al. (2017). For this study, a total of 84 third grade children were assessed. Subdivision of the sample was made based on reading status, where 20 participants were found to be dyslexic while 64 were typical chronologically age matched readers. The average chronological age of the participants was determined to be 8 years and 3 months, ranging from 7 years and 9 months to 8 years and 8 months. All participants were native Dutch-speakers and found to have normal non-verbal IQ, that is, a standardized score ≥80 on the Wechsler Intelligence Scale for Children-III (WISC-III-NL) Block Design subtest (Kort et al., 2005). Based on parental/guardian questionnaires, all participants were found to have no history of brain damage, language problems, psychiatric symptoms, or uncorrected visual or auditory problems.

Similar to Vanvooren et al. (2017), dyslexia status was determined based on evidence of persistent and severe literacy deficits, defined as a score below the 10th percentile on the standardized word reading tasks and/or the spelling task at two consecutive test moments (i.e., in second and third grade). Children who were identified as dyslexic based on a spelling score below percentile 10 also performed below percentile 10 for reading on at least one test moment and below percentile 25 on reading at all test moments. As such, 20 participants in the current sample were found to be dyslexic, while 64 were typical readers.

### Background measures

All participants completed a testing battery to provide a better understanding of the cognitive and literacy skills of each group. All tests were administered in a single session. Descriptive statistics and *t*- and *p*-values from the independent *t*-tests for each background measure are given in Table 2.

Word reading was assessed through the EMT (Brus and Voeten, 1999). This standardized task requires students to read aloud as accurately and quickly as possible a list of 116 Dutch words of increasing difficulty, printed in four columns. The participants were given 1 min to read as many words as possible. The raw score is calculated as the number of words read correctly. The EMT has been found to be a reliable measure (*r* = 0.87), as determined through the use of a parallel test method (Brus and Voeten, 1999).

Pseudo-word reading was assessed by means of the Klepel (Van den Bos et al., 1994). Students are instructed to read aloud as quickly and as accurately as possible a list containing 116 pseudo-words following Dutch grapheme-phoneme correspondence rules. The raw score is calculated as the number of pseudo-words read correctly in 2 min. The Klepel is a standardized test with a reported reliability of *r* = 0.91 determined through the use of a parallel test method.

Phonological awareness was assessed with a phoneme deletion task that has been used previously in reading research in Dutch (Boets et al., 2010) as well as in other populations of a similar age (Hecht et al., 2001). Children are presented with 28 single-syllable non-words and asked to delete a target phoneme of the non-word. The task consists of two parts. The first presents the participant with 10 non-words which results in the production of a real word after deletion of the given phoneme (e.g., DROOS without /d/, an English equivalent would be DROPE without /d/). The second part includes 18 items and results in the production of a non-word after deletion of the given phoneme (e.g., WAPT without /t/). No time restriction is applied. Two practice items are provided before both test sessions to ensure familiarity with task administration. Each correctly solved item is rewarded with one point (maximum = 28). Internal consistency of the test is 0.93 (Evers et al., 2009–2012).

Orthographic Knowledge was measured at the start of grade 3 using a pseudo-homophone task (Bekebrede et al., 2010) consisting of two practice items and 70 test items, visually presented to the child. Bekebrede and colleges reported an internal consistency (Cronbach's alpha) of 0.68. Each item consists of three answer alternatives that are orthographically different, although they are phonologically related (e.g., “voet - voed - foet,” an English equivalent would be “fox - phox - focks”). The child has to determine which alternative is orthographically correct. The orthographic knowledge score is based on the accuracy, with a maximum score of 70.

Four rapid serial naming tasks assessed the rapid serial naming of five familiar colors, objects, numbers and letters (van den Bos et al., 2002). According to Velvis (1998), the average test-retest reliability of the battery is 0.74. For each stimulus type, the child is presented with a card 50 items that are ordered in five columns, randomly arranged. The four tasks were individually administered in the same fixed order: numbers, letters, pictures, and colors. The child was instructed to name the symbols as fast and accurately as possible. The number of correctly identified items per second was recorded. A composite score RAN-Total was created by averaging the z-scores of all four naming tasks.

### Letter-speech sound learning

This study used the same training and assessment procedure as Aravena et al. (2017). All children were provided with a short computer game based training program, aimed at the learning of 8 basic letter-speech sound correspondences within an unfamiliar script (Hebrew), thus allowing for an assessment and comparison between the groups of the initial phase of learning a novel script. Hebrew letters were utilized to remove and control for any influence of prior knowledge, associations, and experience with Dutch orthography. It is believed that by adopting an artificial orthography, such as the one reported in Table 1, a-priori differences in exposure to the experimental stimuli can be controlled for and ruled out.

**Table 1:** Novel letters from Hebrew alphabet and corresponding Dutch speech sounds.

**Letter**	** 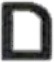 **	** 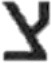 **	** 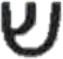 **	** 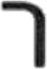 **	** 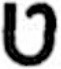 **	** 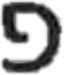 **	** 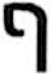 **	** 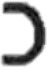 **
Speech sound (IPA)	[a]	[l]	[n]	[t]	[u]	[r]	[k]	[ε]

*IPA, International Phonetic Alphabet*.

The artificial orthography (reported in Table 1) of Aravena et al. (2013, 2017) consists of eight Hebrew graphemes matched to phonemes from the participants' native language, Dutch. The resulting script consists of four vowels and four consonants, with the graphemes presented from left to right. Blomert (2011) noted that the quality of the audio-visual integration of letter-speech sound correspondences in the brain is reflected in the time course of the neural activation of target units and, additionally, manifested at the behavioral level in the associated response latencies during identification. Therefore, a measure of both accuracy and response latencies relating to participants' ability to identify newly learned letter-speech sound correspondences were measured.

#### Training method

Training was carried out with an interactive computer game in which the 8 new letter sound couplings were learned. The objective of the game required children to match target speech sounds presented through headphones, with the corresponding Hebrew symbol that was visually presented on the computer screen inside an animated balloon. The game challenged the children to burst, or pop, the corresponding balloon as quickly and as accurately as possible. Correct associations made the balloon and the surrounding balloons to disappear. If the wrong balloon was popped, no balloons would disappear. This paradigm allowed for the children to learn the correct couplings through trial and error, thus allowing for implicit learning. When all balloons had been popped and disappeared, the child moved on to the next “balloon field.” The clearing of several “balloon fields” allowed for the advancement to the next level which advanced in the level of difficulty through the addition of extra letters in the balloon field, advancing from 2 to eventually 8 different letters. The faster the child played, the more points were awarded. Each child was trained for 20 min per session, regardless of the level of difficulty attained.

#### Letter-speech sound identification within the artificial orthography

The Lexy association test was administered directly after training. Each child was presented target Dutch speech sounds through a headphone and was instructed to select the corresponding Hebrew letter from two alternatives presented on a computer screen (50% chance level). All presented Hebrew letters were the same as those used in the training game. Each child was instructed to indicate as fast and as accurately as possible which sign corresponds to the sound they heard, allowing for measures of both accuracy and speed of access to the learned couplings. A maximum correct score of 56 was achievable. Speed was measured in milliseconds per coupling allowing for the creation of a Lexy rate score of average time (milliseconds) per correct item.

#### Reading within the artificial orthography

The 3MAST, a reading task within the artificial orthography was administered following the Lexy association measure, consisting of 22 high-frequency Dutch words written within the artificial orthography. Each word consisted of two to four Hebrew letters and was presented in two columns of 11 words on a printed card. The child was provided 3 min to read as many of the words as possible aloud. Similar to the EMT and Klepel, a rate score (words per second) was calculated representing the number of correctly read words within 3 min.

### Statistical analyses

Statistical analyses were performed with SPSS 20.0 software (IBM Corp., 2011). All variables were found to be normally distributed as checked within each group by the Shapiro-Wilk's test for normality (*p* > 0.05) with the exception of the accuracy score of the Lexy and 3MAST rate measure. To approach a normal distribution, the Lexy accuracy score was transformed by a reflect logarithmic transformation that led to a distribution that was found to be normal and so the transformed scores were used in the analyses. Due to the lack of normality achieved through the application of various transformations of the 3MAST task, nonparametric tests were utilized in the analysis of results relating to this variable. Homogeneity of variance was assessed by Levene's Test for Equality of Variances. Group comparisons were investigated based on an independent samples *t*-test for all measures with the exception of the 3MAST task. Correction for multiple testing was applied across all group comparisons to avoid the likelihood of false positive conclusions through the application of the False Discovery Rate (FDR) procedure, a simple sequential Bonferroni-type procedure that has been proven to control for the false discovery rate for independent test statistics (Benjamini and Hochberg, 1995). Concurrent relations between measures of orthographic knowledge, phonological awareness, RAN, reading and outcome measures of the letter-speech sound learning tasks were evaluated with Pearson and Spearman correlations.

To assess the portion of the unique variance of reading and non-word reading explained by letter-speech sound binding, a series of stepwise linear regression analyses were calculated across both groups. For each model, reading and non-word reading performance was predicted by measures of phonological awareness (PA), orthographic knowledge (Ortho), RAN, and the two letter-speech sound learning and binding outcome measure: Lexy and 3MAST.

Prior to conducting a hierarchical multiple regression, assumption testing revealed the presence of an adequate sample size (*n* = 84) given the inclusion of five independent variables included in the analysis (Tabachnick et al., 2001). The assumption of singularity was also met as the independent variables (phonological awareness, orthographic knowledge, RAN, Lexy and 3MAST) were not a combination of other independent variables. An examination of correlations (see Table 3) revealed that no independent variables were highly correlated, except orthographic knowledge with both phonological awareness and RAN. However, as the collinearity statistics indicate that the variance inflation factors (VIF) were all within acceptable limits (all VIFs < 1.67), the assumption of multicollinearity was deemed to have been met (Hair et al., 1998). Histogram and P-P plots of the standardized residuals for each model and scatter plots indicated the assumptions of normality, linearity, and homoscedasticity were all satisfied (Hair et al., 1998).

## Results

### Differentiating typical readers and individuals with dyslexia

Similar to past studies typical and dyslexic readers were found to differ across all cognitive measures of phonological awareness, orthographic knowledge and rapid naming in addition to both literacy measures, as reported in Table 2.

For the purpose of examining whether the brief letter-speech sound training could differentiate between participants with or without dyslexia, groups were compared across all letter-speech sound learning related measures. Mean scores and tests statistics for each comparison are also reported in Table 2. Results indicate that both groups performed equally well on both speed and accuracy measures with regard to novel letter naming *p*s > 0.400. On the other hand, individuals with dyslexia were on average found to underperform when having to apply the newly learned novel script in the 3MAST word reading task *p* < 0.001. Analysis of the effect size utilizing eta-squared values indicated that nearly 17% of the variability in the reading rate of an artificial orthography could be accounted for by the individual's reading status.

**Table 2:** Performance and group comparisons on literacy and cognitive tasks.

	**Control (*****n*** = **64)**		**Dyslexic (*****n*** = **20)**				
**Measure**	***M***	***SD***	***M***	***SD***	***T***	***p***	**Cohen's *d***
Word reading	46.0	12.8	22.5	8.6	7.641	< 0.001^*^	2.155
Non-word reading^∧^	38.3	12.3	15.7	6.6	10.560	< 0.001^*^	2.289
Phonological awareness	20.0	5.1	13.7	5.4	4.813	< 0.001^*^	1.200
Orthographic knowledge	45.0	8.7	30.75	5.3	6.883	< 0.001^*^	1.978
RAN-Total	1.4	0.2	1.2	0.2	5.885	< 0.001^*^	1.500
Lexy Accuracy	51.0	5.8	50.7	5.0	0.133	0.894	0.055
Lexy Rate	2,282	593	2,404	632	0.790	0.432	0.199
3MAST^∧^	0.05	0.04	0.01	0.01	273.0^a^	< 0.001^*^	0.171^b^

^ *failed Levene's test for Equality of Variance*.

* *significant p-value after applying the FDR procedure*.

a *Mann-Whitney U test*.

b *eta-squared, η^2^*.

### Correlation analysis

To determine the concurrent relations between measures of letter-speech sound learning within the artificial orthography, phonological awareness, orthographic knowledge, RAN, word and non-word reading, Pearson and Spearman correlations were calculated and are reported in Table 3.

**Table 3:** Pearson correlations between measures of literacy and cognitive measures.

**Measures**	**2**	**3**	**4**	**5**	**6**	**7**	**8^∧^**
1. Word reading	0.911^***^	0.677^***^	0.815^***^	0.525^***^	0.086	0.027	0.315^**^
2. Non-word reading	–	0.701^***^	0.776^***^	0.407^***^	0.044	0.022	0.279^*^
3. Phonological awareness		–	0.685^***^	0.303^**^	0.145	0.004	0.306^**^
4. Orthographic knowledge			–	0.329^**^	0.125	0.108	0.415^***^
5. RAN-total				–	0.124	0.113	0.056
6. Lexy accuracy					–	0.437^***^	0.584^***^
7. Lexy rate						–	0.268^*^
8.3MAST^∧^							–

* *p < 0.05*.

** *p < 0.01*.

*** *p < 0.001*.

∧ *Spearman correlations*.

As was expected, phonological awareness, orthographic knowledge, and RAN were found to be significantly correlated with both word and non-word reading. The 3MAST measure of reading within the artificial orthography was found to correlate significantly with letter knowledge accuracy and rate in the novel script. In addition, the 3MAST task was found to correlate significantly with phonological awareness, orthographic knowledge, word and pseudoword reading, but not with RAN. The Lexy scores were not found to correlate with any of the assessed variables with the exception of the 3MAST task. When IQ and age were introduced across all subjects to control for any spurious effects, all relations were maintained.

### Predicting individual differences in literacy achievement

To test the unique variance of reading explained by each of the predictor variables a series of hierarchical multiple regression analyses were conducted with word reading and non-word reading as the dependent variables. All independent variables were entered at stage one of the regression with the exception of the target independent variable. This was done to allow for the assessment of the unique contribution of each independent variable in explaining individual variance in word and non-word reading above that of the additional controls. Inter-correlations between the hierarchical multiple regression variables were reported in Table 3, and the regression statistics are in Table 4.

**Table 4:** Unique variance in word and non-word reading, accounted for by measures of letter-speech sound binding (3MASTRate), phonological awareness (PA), orthographic knowledge and rapid naming (RAN-Total) (*R*^2^change and standardized Beta).

	**Word reading**		**Non-word reading**	
	***R*^2^ change**	**β**	***R*^2^ change**	**β**
1a. Phonological awareness	0.012^*^	0.206	0.038^**^	0.274
1b. Orthographic knowledge	0.177^***^	0.640	0.150^***^	0.589
1c. RAN-total	0.054^***^	0.255	0.010	0.111
1d. 3MAST	0.000	0.002	0.003	0.061
Total *R*^2^	0.770		0.688	

* *p < 0.05*.

** *p < 0.01*.

*** *p < 0.001*.

The full model of PA, orthographic knowledge, RAN, Lexy accuracy and 3MAST to predict word reading was statistically significant, *R*^2^ = 0.770, *F*_(4, 76)_ = 63.693, *p* < 0.001, adjusted *R*^2^ = 0.758. Similarly the full model predicting non-word reading was found to be statistically significant, *R*^2^ = 0.688, *F*_(5, 76)_ = 41.814, *p* < 0.001, adjusted *R*^2^ = 0.671.

Results revealed that the addition of PA to the prediction of word reading (after controlling for any variance explained by the other independent variables) led to a statistically significant increase in *R*^2^ = 0.012, *F*_(1, 76)_ = 4.065, *p* = 0.047. Similarly, orthographic knowledge and RAN were found to uniquely contribute to word reading, with respectively *R*^2^ = 0.177, *F*_(1, 76)_ = 58.655, *p* < 0.001 and *R*^2^ = 0.054, *F*_(1, 76)_ = 17.721, *p* < 0.001.

The hierarchical multiple regression analysis with non-word reading as a dependent variable found that PA [*R*^2^ change = 0.038, *F*_(1, 76)_ = 9.357, *p* = 0.003], and orthographic knowledge [*R*^2^ change = 0.150, *F*_(1, 76)_ = 36.549, *p* < 0.001] each uniquely contributed to a statistically significant increase in *R*^2^ when entered as the independent variable in the model. While RAN-Total was not found to offer and additional contribution [*R*^2^ change = 0.010, *F*_(1, 76)_ = 2.464, *p* = 0.121].

Although the children's reading within the artificial orthography, as measured with the 3MAST task, was found to be significantly related to word and non-word reading the addition of the 3MAST task in the model found that reading in the newly learned artificial orthography did not offer any additional contribution to explaining the variance of word and non-word reading once measures of PA, orthographic knowledge and RAN were controlled for. Therefore, results of this analyses could not support our research hypothesis which predicted a significant independent contribution of our measure of letter-speech sound binding on reading outcomes.

## Discussion

This study examined letter-speech sound learning using a novel script (Hebrew) paired with speech sounds taken from the participants' native language (Dutch). Both groups of children with dyslexia and chronologically age matched controls, underwent a 20-min training program administered with the aim of teaching them eight basic letter-speech sound correspondences within a novel orthography. Assessments of both mastery of identification and word reading in the novel script were made. Additionally, this study set out to investigate letter-speech sound learning's relation with measures of phonological awareness, orthographic knowledge and RAN as well as the combined and unique contribution these variables have on reading and non-word reading achievement. Supporting past research, our predictor variables of phonological awareness, orthographic knowledge and RAN were found to be related to both reading outcomes of word and non-word reading. Additionally, our measure of reading within the artificial orthography was found to correlate significantly with word and non-word reading.

Results from this study demonstrated that most children in both groups were capable of mastering the new letter-speech sound correspondences within the allotted time by the use of our implicit learning game. As such, groups were not found to differ significantly on the measure of grapheme-phoneme learning, thus we could not support past findings reporting differences between children with and without dyslexia when matching, under time pressure, graphemes of an artificial-letter script with phonemes (Aravena et al., 2013, 2017; Karipidis et al., 2018). However, outcome measures assessing the child's ability to apply these newly learned correspondences to decode text were found to significantly differ between the reading groups (dyslexic vs. typical readers). These results replicate Aravena et al. (2013, 2017) findings of the presence of group differences in word reading rate measures within the novel script (3MAST rate). Aravena et al. (2013) argued that the presence of such group differences suggests a difficulty in the early stages of cross-modal integration of phonemes and graphemes in children with dyslexia. Aravena et al. (2013, 2017) went on to suggest that a deficit in the process of cross-modal integration of phonemes and graphemes is a key contributing factor in the expressed literacy difficulties of individuals with dyslexia, and could be invoked at any time by the presentation of a novel script, as was done in the 3MAST task. However, it must be noted that our interpretation of these results and our support of Aravena and colleagues' acieration is limited as the 3MAST task was administered directly after a short training period and, therefore, cannot be said to be a direct measure the integration of letter-speech sound correspondences, as a greater amount of training time would be required. Although the 3MAST task is believed to offer an assessment of an individual's ability to instrumentally use newly learned letter-sound correspondences, it should be noted that this task additionally relies partly on additional processes such as verbal short-term memory and phonological awareness skills (i.e., phoneme blending), thus, resulting in ambiguity surrounding our interpretation of the 3MAST task as a measure of the early stages of letter-speech sound learning, especially since the expected differences in the Lexy task were not observed. Additionally, given that performance on the 3MAST task was found to be correlated with existing reading ability, the directionality of the relationship could be argued in such a way that performance on the 3MAST task is a consequence of reading impairment or experience and not a cause. Results of the regression analyses demonstrated that our measure of reading in an artificial script made no meaningful contribution in explaining individual differences in reading and non-word reading, after controlling for phonological awareness and orthographic knowledge, indicating that the uncontrolled contribution of the 3MAST task in explaining reading outcomes is a function of unimodal phonology and orthography processing.

However, since our study involved the learning of an artificial orthography, it was believed and argued by Aravena et al. (2013, 2017) that any group differences in reading experience would have been controlled for. As an artificial orthography was used, a relation between our Dutch measure of orthographic knowledge and reading in an artificial orthography was not expected, yet observed. One possible explanation for this relation may be found in reading experience's impact on brain development. Neurodevelopmental studies of children and ex-illiterates have demonstrated a clear neurological development in response to reading experience (Dehaene et al., 2010; Thiebaut de Schotten et al., 2014). Findings have shown that as reading improves an increased unilateral activation in the left occipito-temporal region or visual word form area is observable (Shaywitz et al., 2002). Additionally, learning to read does not merely alter an individual's visual cortex response to written words. Studies have revealed measurable changes in the language areas of the left hemisphere associated with phoneme perception and articulation (Turkeltaub et al., 2003). Therefore, it could be argued that regardless of the specific control for past reading experience, provided by our novel script reading measure, differences in the brain circuitry caused by individual differences in literacy experience may still exhibit themselves as a more optimal means of processing newly learnt orthographic units and/or phoneme articulation and blending which is also required when reading in the artificial orthography.

Furthermore, Nash et al. (2017) demonstrated that children with dyslexia's degree of letter-sound integration was appropriate for their reading level, suggesting that compromised letter-speech sound integration may be a function of reading level. However, in contrast to our design, the study of Nash and colleagues used a grapheme-phoneme task based on the participant's native language, not an artificial one which has been argued to remove the influence of past orthographic knowledge (also see Blomert and Willems, 2010). Thus, ambiguity still exists concerning the directionality of the relationship, as our research design limits our interpretations of such cause and effects.

## Conclusion

The present data cannot directly support the notion of a letter-speech sound integration deficit in dyslexic children. However, findings do indicate that children with dyslexia are less well able to use newly acquired phoneme-grapheme rules to decode words. As discussed above, this is most likely a result of reduced reading experience/level and differences in the phonological skills required for decoding. Our study was not able to support letter-speech sound learning as an independent contributor to reading difficulties of individuals with dyslexia. However, our findings are in line with past research that has implicated impaired phonological awareness as the source of reading impairment (Ramus et al., 2003; Law et al., 2017a).

In summary, our results appear to suggest that within a short period of training dyslexics can learn to identify the letter speech sound combination. However, when applying it, failure occurs. This could be argued to be due to either (1) other reading-related processes which are needed to perform this task, such as phonological skills, RAN or verbal short-term memory, or specific neurological changes related to increases print exposure, or (2) based on the rational of Aravena et al. (2013, 2017) the lack of full integration of, or access to, the new grapheme-phoneme correspondences can be argued to be essential when having to apply these correspondences within a reading task (also see Van Atteveldt et al., 2004). Based on the evidence provided within this study and for reasons outlined within the discussion we feel the best explanation for the pattern of results reported in this paper lays with the influence of past reading experience and reading related processes, and not with the lack of full integration of, or access to, the new grapheme-phoneme correspondences.

## Ethics statement

This study has been approved by the KULeuven Research Ethics Committee. Written informed consent was obtained from all parents and/or guardians of each participating child included in the study.

## Author contributions

All listed authors contributed to the editing and preparation of this manuscript. JL was the lead author in charge of producing the intel drafts of the manuscript along with conducting and reporting the included data analysis. AD, JV, and MV carried out and supervised the data collection and subject recruitment. MV was responsible for the design and construction of the study and tasks. JW and PG inanition to providing support during the writing process offered guidance and support throughout all stages from design, data collection and analysis of the study.

### Conflict of interest statement

The authors declare that the research was conducted in the absence of any commercial or financial relationships that could be construed as a potential conflict of interest.
